# Volcanic ash as a driver of enhanced organic carbon burial in the Cretaceous

**DOI:** 10.1038/s41598-018-22576-3

**Published:** 2018-03-08

**Authors:** Cin-Ty A. Lee, Hehe Jiang, Elli Ronay, Daniel Minisini, Jackson Stiles, Matthew Neal

**Affiliations:** 1 Department of Earth, Environmental and Planetary Sciences, Rice University, Houston, TX USA; 2 Department of Earth and Environmental Sciences, Vanderbilt University, Nashville, TN USA; 3 Shell International Exploration and Production Inc., Shell Houston Technology Center, 3333 Highway 6 South, Houston, Texas 77082 USA

## Abstract

On greater than million year timescales, carbon in the ocean-atmosphere-biosphere system is controlled by geologic inputs of CO_2_ through volcanic and metamorphic degassing. High atmospheric CO_2_ and warm climates in the Cretaceous have been attributed to enhanced volcanic emissions of CO_2_ through more rapid spreading at mid-ocean ridges and, in particular, to a global flare-up in continental arc volcanism. Here, we show that global flare-ups in continental arc magmatism also enhance the global flux of nutrients into the ocean through production of windblown ash. We show that up to 75% of Si, Fe and P is leached from windblown ash during and shortly after deposition, with soluble Si, Fe and P inputs from ash alone in the Cretaceous being higher than the combined input of dust and rivers today. Ash-derived nutrient inputs may have increased the efficiency of biological productivity and organic carbon preservation in the Cretaceous, possibly explaining why the carbon isotopic signature of Cretaceous seawater was high. Variations in volcanic activity, particularly continental arcs, have the potential of profoundly altering carbon cycling at the Earth’s surface by increasing inputs of CO_2_ and ash-borne nutrients, which together enhance biological productivity and burial of organic carbon, generating an abundance of hydrocarbon source rocks.

## Introduction

**Figure 1:**
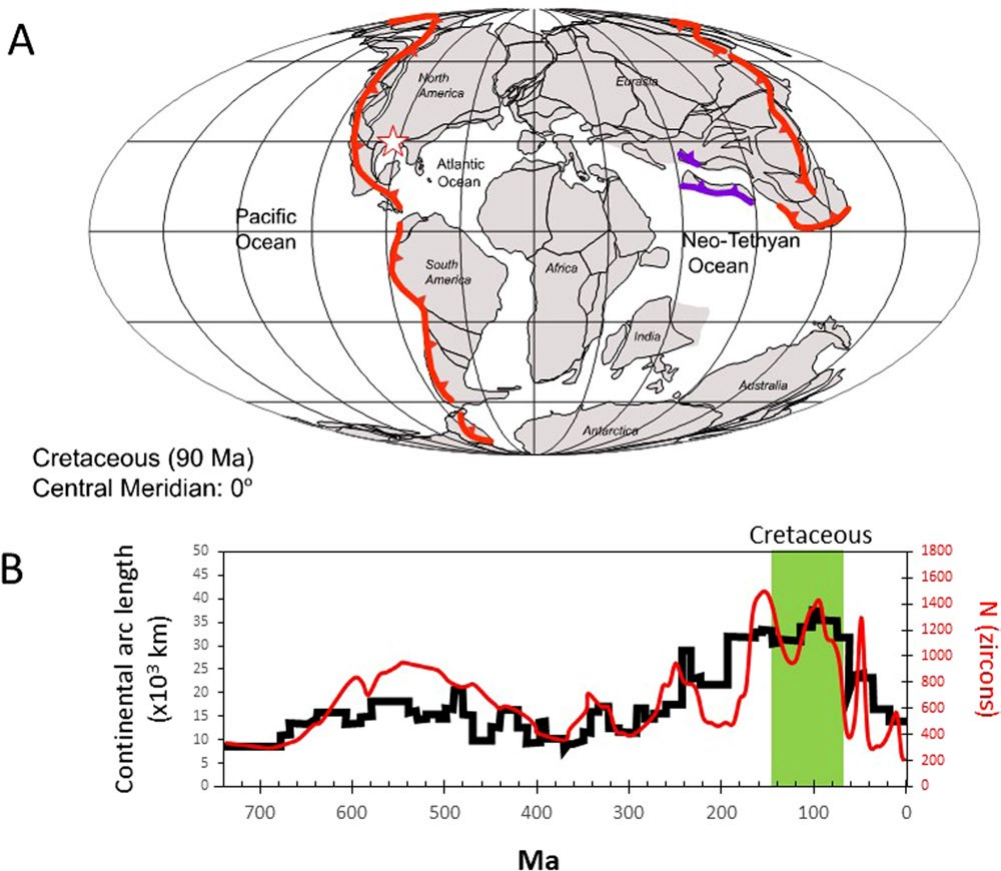
(**A**) Extent of continental magmatic arcs (red lines) above ocean-continent subduction zones in the Cretaceous map taken from Cao *et al*.^23^. Location of field site DR11 shown by star. (**B**) Length of continental arcs (black line, left vertical axis) and detrital U/Pb zircon ages (red line, right vertical axis) through time compiled from the literature^23,94^. Continental arc length is based on compilation of granitoid plutonic belts through time^23^ and does not necessarily correspond with total subduction lensgth.

The Cretaceous period (145–66 Ma) was characterized by unusually warm (greenhouse) climate with little or no permanent polar ice caps, high (eustatic) sea level and atmospheric CO_2_ concentrations more than ten times higher than that of today^1–9^. The Cretaceous was also characterized by high burial rates of carbon in the form of carbonates and organic carbon, much of the latter forming a significant proportion of the world’s hydrocarbon source rocks^10–15^. The high atmospheric CO_2_ concentrations and carbon burial fluxes suggest that geological inputs of CO_2_, through volcanism and metamorphism, were high^13,16–22^. These high CO_2_ inputs are widely thought to have been supported by high mid-oceanic ridge spreading rates and a high frequency of flood basalts^13,19–22^. However, the length of continental arcs may have also been longer (>2 times) in the Cretaceous than during the Cenozoic (Fig. 1A,B), leading to suggestions that inputs of CO_2_ into the atmosphere from interaction of continental arc magmas with ancient carbonates stored in the continental plate may have also been important^16,17,23–27^.

## Ash in the Cretaceous Western Interior Seaway

Continental arc magmas, due to their higher volatile contents and more silicic compositions, tend to be more explosive than island arc lavas^28^. Volcanic ash is abundant in sediments of the Cretaceous Interior Seaway in western North America, manifesting as numerous discrete layers correlative over hundreds of kilometers^29,30^. The source of such ash came from the continental magmatic arc to the west (Fig. 1A), e.g., the Peninsular Ranges, Sierra Nevada and Idaho batholiths, associated with eastward subduction of the Farallon oceanic plate beneath western North America^31,32^. Experimental studies as well as studies of modern ash dispersal and deposition have shown that ash, largely composed of highly unstable and reactive volcanic glass, alters quickly and can become an important source of nutrients for life^33–42^. There are recent cases of individual modern volcanic eruptions, which were followed by local phytoplanktonic blooms in marine and lacustrine environments^43–50^. However, there is considerable variability in the reactivity of ash, with basaltic ash being more reactive than more silicic ashes^39^. These modern observations raise the question of whether globally enhanced ash production in the Cretaceous may have extensively fertilized the ocean.

## Geochemistry of Cretaceous ashes

**Figure 2:**
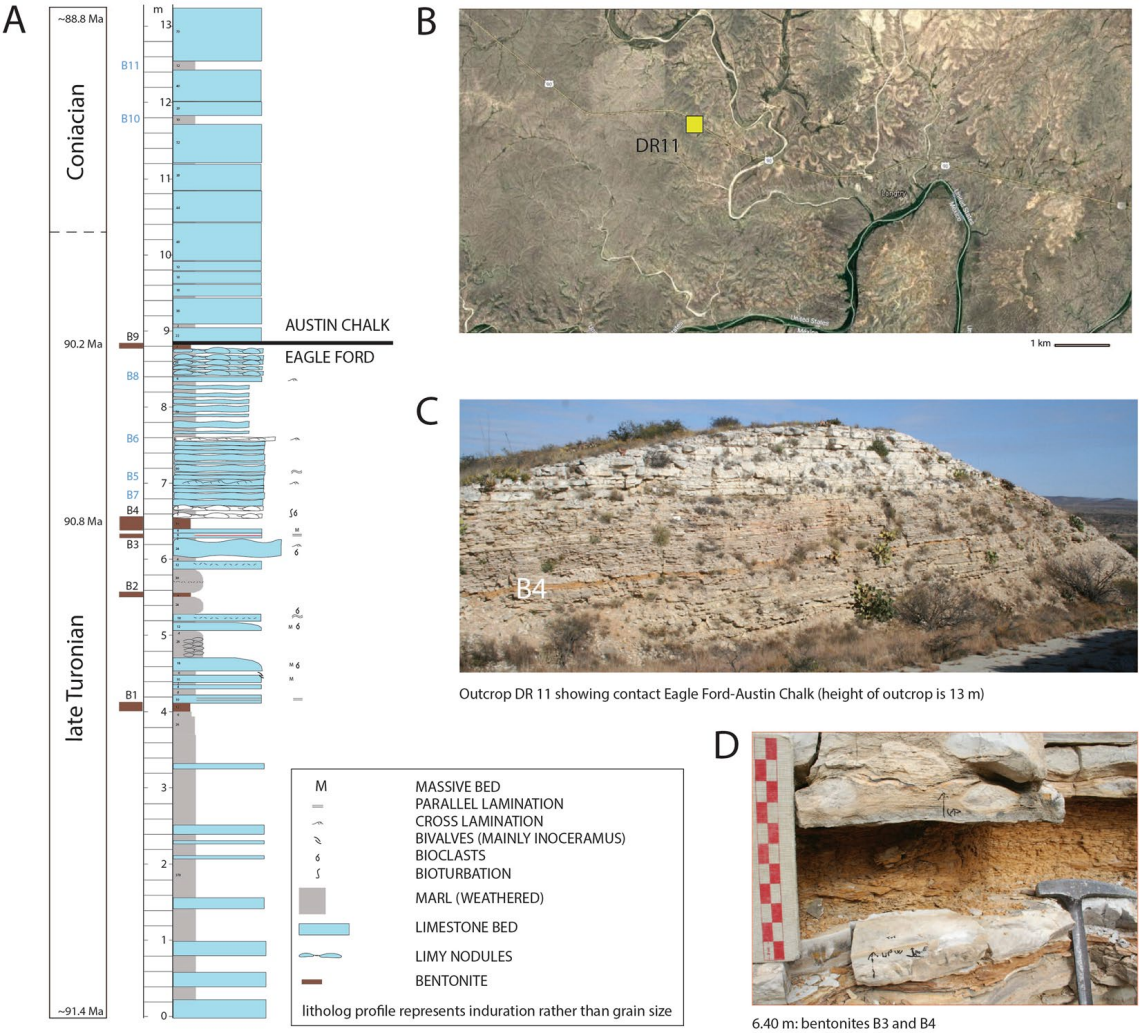
Field site in west Texas. (**A**) Stratigraphic section of outcrop DR11 modified from Minisini *et al*.^56^. Discrete ash layers noted as brown and denoted with black font in the stratigraphic column, while marls with disseminated ash are noted in blue font. (**B**) Exact location of outcrop DR11. Image is from Google Earth (© Google, DigitalGlobe, CNES/Airbus, Map data © Google, INEGI). (**C**) Outcrop photo with ash horizons appearing as orangish recessed layers. (**D**) Discrete ash layer B4 (thick) is shown for reference and refers to the thickest layer seen in the outcrop in C.

Ash beds are unfortunately almost always highly altered to clays (e.g., bentonite) and bear little resemblance to their original unaltered protoliths save for a few relict grains of volcanic minerals^51^. Here, we present a method for estimating the unaltered protolith composition, allowing us to quantify what fraction of life-essential elements may have been leached. We focus on Si, Fe, and P because Si is important for diatomaceous organisms and because Fe and P are considered essential nutrients^52^. We investigated the Upper Cretaceous (middle to late Turonian) marlstones and limestones from the uppermost Eagle Ford and lowermost Austin Chalk formations in southwest Texas (Fig. 2). The Eagle Ford and Austin Chalk groups are characterized by numerous discrete ash horizons^53–56^ much like similar aged sediments in the Bighorn Basin in Wyoming^31,32^. The Eagle Ford and Austin Chalk are represented by finely laminated marlstones, deposited in a distal portion of the interior seaway and starved of significant clastic sedimentary inputs^53,56,57^. The Eagle Ford, in particular, is characterized by high organic carbon contents and is an economically important hydrocarbon source rock. Recent geochronology and chronostratigraphy studies have shown that despite the distal environment, sedimentation rates were high (averaging 10–20 m/My and up to 60 m/My) due to high rates of biogenic carbonate sedimentation^54–56^.

We sampled altered ash from the uppermost Eagle Ford and lowermost Austin Chalk formations from a ~13 m vertical section exposed in a roadcut (29.826030°, −101.623631°) along old highway 90 near Osman Canyon, Texas, USA^53,56^. This is the same outcrop described in Minisini *et al*. as DR11^56^ and is situated next to a 100 m core investigated in the same study (INNES-1). Based on chronostratigraphy from this outcrop and adjacent core^56^ and of a nearby section in Lozier Canyon, the age of this section is around 90–91 Ma^54,55^. Ash layers were identified in the field by their orange color and tendency to form recessed layers between more resistant limestone and marlstone layers. We analyzed discrete ash horizons ranging in thickness from <0.5 up to 10 cm as well as some marls. Ash layers are laterally continuous for 100 s of meters along the entire width of the outcrop. The thickest ash layers are clay-rich (illite, montmorillonite, kaolinite), but the thin ash layers can be diluted by diagenetic carbonate. Although the ashes have been completely altered to clays, fresh euhedral quartz and zircon grains of volcanic origin are present. Rounded quartz or zircon grains of detrital origin are not observed, indicating that the ash beds have not been contaminated or diluted by clastic sediments.

We analyzed ash horizons using laser ablation inductively coupled plasma mass spectrometry. For each ash horizon, we typically analyzed five, randomly spaced 110 µm diameter ablation spots (see Methods) from polished thick sections. Because of local heterogeneity, we averaged these ablation shots to obtain an average composition for the ash. For comparison, we have also compiled bulk rock compositions of ash layers from nearby localities of similar age in the Eagle Ford, lower Austin Chalk, and Del Rio formations, the latter underlying the base of the lower Eagle Ford^58,59^.

## Ash protolith compositions and chemical depletion factors

**Figure 3:**
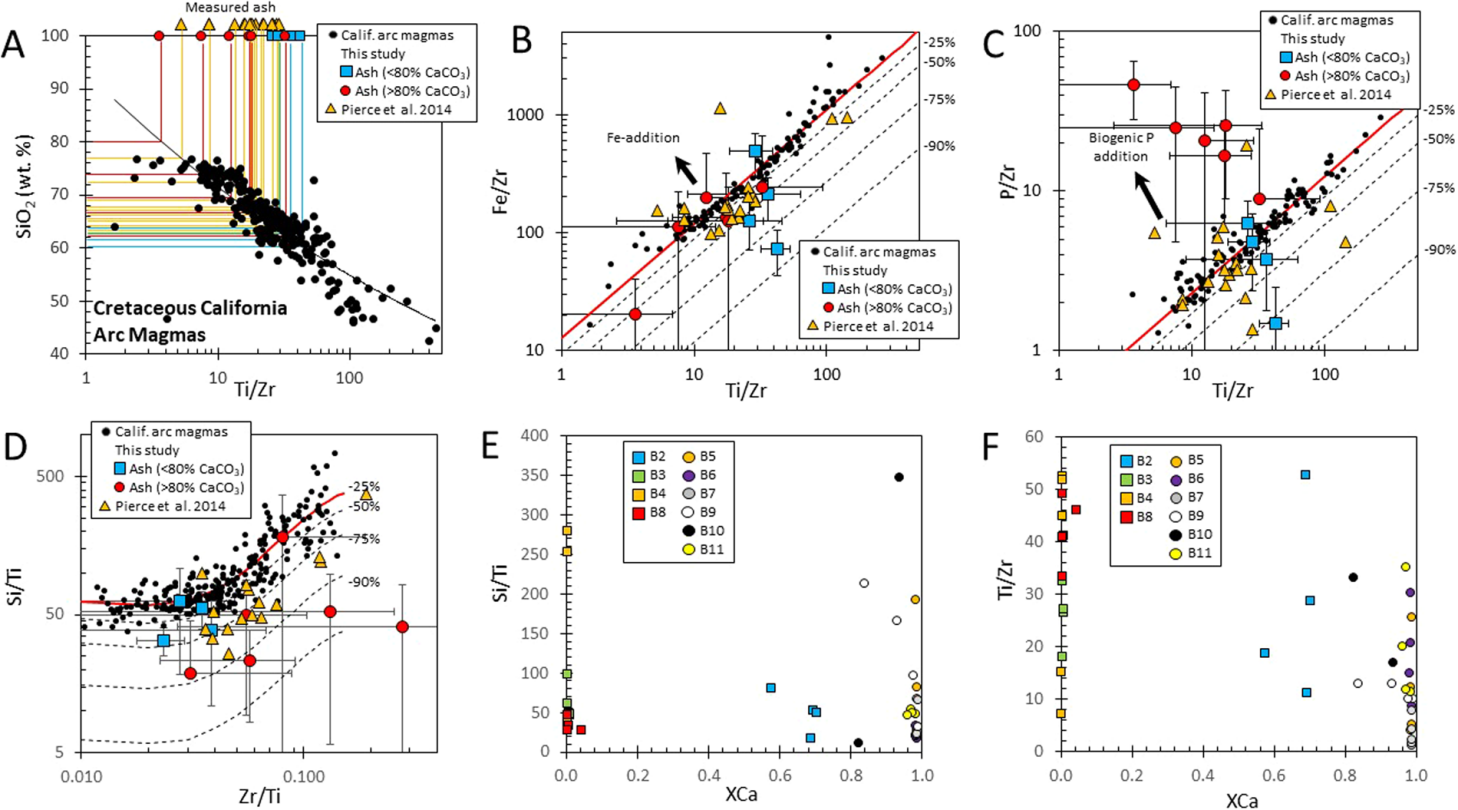
(**A**) Plutonic rock data (black circles) from the Cretaceous Peninsular Ranges Batholith in California^65^ plotted as SiO_2_ (wt. %) versus Ti/Zr (by weight). Red circles (thin, carbonate-rich layers) and blue squares (thick, carbonate-poor layers) represent measured Ti/Zr of ash layers. Horizontal red, orange and blue lines with arrows represent approximate SiO_2_ content of protolith based on empirical correlation (curved black line) of SiO_2_ versus Ti/Zr from the batholith data. (**B**–**D**) Average Fe/Zr and P/Zr versus Ti/Zr and Si/Ti versus Ti/Zr (all ratios by weight). Altered ash is given by red circles and blue squares and unaltered batholith rocks by gray open circles from this study. Curved lines represent regressions with details given in the text. Error bars represent one standard deviation. (**E** and **F**) Si/Ti and Ti/Zr (by weight) versus XCa, which is the mass fraction of Ca relative to the total mass of cations (i.e., cation mass fraction), a measure of carbonate fraction. Samples represent individual measurements, in contrast to (**B**–**D**), where only the average for each sample is shown. Blue squares in (**E** and **F**) represent thick, carbonate-poor ash horizons and red circles represent thin, carbonate-rich ash layers. Orange triangles in (**A**–**D**) represent bulk rock samples of ash horizons from^58,59^; standard deviations are not provided for these samples because analyses represent only one bulk rock measurement.

Ash layers are rich in Na, K and Al, and low in Si and Fe compared to average continental crust as a result of the high clay content of the ashes. Negative correlations with Ca and Sr are due to the diluting effects of variable amounts of calcium carbonate. Of interest is how much Si, P and Fe has been depleted from the original unaltered ash and what the composition of the unaltered protolith was. We use the relative abundances of Ti and Zr because they are relatively immobile during aqueous alteration^60^ and because they exhibit distinct behaviors during magmatic differentiation in continental arcs^61,62^. During differentiation of continental arc magmas, Si and Zr increase due to fractionation of mafic minerals which are poor in Si and largely devoid of Zr. At extreme differentiation, Zr decreases slightly owing to a decrease in zircon solubility^63^. Fractionation of magnetite, a feature of calc-alkaline series differentiation common to subduction zone magmatism, removes Ti and Fe during differentiation^64^. P increases early during differentiation but decreases as magmas become more silicic. These systematics can be seen in Fig. 3A–D, where we have plotted the compositions of plutonic rocks from the Cretaceous Peninsular Ranges batholith in southern California^65^, which are similar to that of other continental arcs^61^. Magmatic differentiation leads to monotonic increase in SiO_2_ with decreasing Ti/Zr (Fig. 3A), Fe/Zr versus Ti/Zr (Fig. 3B), P/Zr versus Ti/Zr (Fig. 3C), and Si/Ti versus Zr/Ti (Fig. 3D) with the following empirical relationships (applicable only to magmas with molar Mg/(Mg + Fe) <0.65(: SiO_2_ (wt. %) = 93.02(Ti/Zr)^−0.134^ (r^2^ = 0.70), Fe/Zr = 12.582(Ti/Zr)^0.9689^ (r^2^ = 0.94), P/Zr = 0.4279(Ti/Zr)^0.7299^ (r^2^ = 0.90), and Si/Ti = 77.2–2.024 × (Zr/Ti) + 5.739 × 10^4^(Zr/Ti)^2^–2.026 × 10^5^(Zr/Ti)^3^ (r^2^ = 0.62) (Si/Ti versus Zr/Ti equation is valid only between 0.01–0.2 Zr/Ti). In Fig. 3E,F, we also show that the above chosen element ratios do not correlate with indices of carbonate, such as Ca, indicating that Si, Fe, Ti, and Zr derive from the altered ash component. A significant exception is high P/Zr in carbonate-rich samples because of the higher P content of carbonates and the very low amounts of Zr in carbonates (Fig. 3C).

We estimate the mass fraction of Si, P or Fe leached (depletion factor) from altered ash by comparing immobile-element normalized Si, P and Fe ratios to immobile element ratios in ash and the above empirical magmatic differentiation arrays (Fig. 3A–D). For example, the Si depletion factor is determined by assuming Ti is perfectly immobile:

1 $$\frac{{M}_{Si}^{L}}{{M}_{Si}^{o}}=1-(\frac{{C}_{Si}^{re}}{{C}_{Ti}^{re}})(\frac{{C}_{Ti}^{o}}{{C}_{Si}^{o}})$$

The left hand side represents the Si depletion factor, where ${M}_{Si}^{o}$ is the original mass of Si in the rock and ${M}_{Si}^{L}$ is the total mass of Si lost. The quantities ${C}_{Si}^{re}$ and ${C}_{Ti}^{re}$ are the concentrations of Si and Ti remaining in the altered ash (equivalent to the measured concentration), ${C}_{Si}^{o}$ and ${C}_{Ti}^{o}$ are the concentrations of Si and Ti in the original rock, and ${C}_{Si}^{re}/{C}_{Ti}^{re}$ is the Si/Ti ratio of the altered ash and ${C}_{Si}^{o}/{C}_{Ti}^{o}$ is the Si/Ti ratio of the unaltered protolith. Graphically, Si-depleted ashes would plot below the magmatic array in a Si/Ti versus Zr/Ti diagram (Fig. 3D).

Based on the Ti/Zr systematics of ash from this study and from^58,59^, most of the ashes are found to have andesitic protoliths with an average SiO_2_ content of 63 ± 6(1σ) wt. % SiO_2_ (Fig. 3A; Supplementary Tables). Interestingly, the ash protolith is similar to the average SiO_2_ content of the Peninsular Ranges Batholith (64 wt. %)^65^. Most of the ashes are depleted in Fe and Si with depletions up to 70% (Fig. 3B,D; Supplementary Tables). Si is consistently depleted, but Fe shows some enrichments, possibly due to oxidation of pyrite (which is locally common in the section) and subsequent diffusion of Fe into to the ash. Calculated Fe depletion factors are thus minimum bounds. For P, we see that the thick ash beds are depleted up to 75%. However, there is extensive P addition (organic P) associated with carbonate-rich sediments. We thus ignore the P contents of those with >80% carbonate. In summary, the Si, Fe, and P depletion factors are much larger than depletions associated with chemical weathering of igneous or metamorphic basement, as inferred from the geochemistry of soil profiles or clastic sediments^60^. However, these extreme chemical depletions should not seem surprising as it has been shown that volcanic glass alters rapidly in seawater^36^.

We speculate that most of the leaching occurred during early diagenesis. If silica had been leached well after burial, we might expect to see re-precipitation of silica elsewhere in the system, but only limited silicification, such as cherts, is found in the section. This suggests that any Si loss was transported into the ocean during early diagenesis rather than re-distributed deep within the sedimentary column. Indeed, sedimentary pore waters typically become saturated in Si at depths greater than ~0.5 m depth^66^, which would also suggest early Si loss. Using the reported average sedimentation rates of 10–20 m/My and assuming most Si loss occurred within the upper 0.5 m of the sediment, we estimate an individual ash layer to contribute to a sediment to seawater flux of Si over a 25–50 ky interval. Exactly how much is released during settling through the water column compared to during burial diagenesis is unclear. Regardless, the high abundance of ash layers in the Eagle Ford suggest ash fall recurrence intervals of less than 10 ky^54–56^, which being shorter than the burial diagenesis time of 25–50 ky, suggests that the ash-to-seawater flux of dissolved Si, P and Fe by alteration may have been somewhat continuous during the interval of arc magmatism, even if ash events themselves are episodic.

## Global ash-laden nutrient fluxes

We now evaluate the potential global effect of a flare-up of continental magmatic arcs in the Cretaceous. Global nutrient inputs (moles/y) associated with windblown ash from continental arcs can be estimated with the following equation, $Lw\dot{M}\rho {C}_{i}\,{f}_{ash}({M}_{i}^{L}/{M}_{i}^{o})$, where *L* is the length (km) of continental arcs *in excess of the present*, *w* is the average width of the magmatic arc (km), $\dot{M}$ is the magmatic flux (*km*^3^*/km*^2^*/My*), *ρ* is average crustal density (2800 kg/m^3^), *C*_*i*_ is the concentration of an element *i* in an average continental arc, *f*_*ash*_ is the mass fraction of the magmatic flux that erupts in the form of ash, and ${M}_{i}^{L}/{M}_{i}^{o}$ is the mass fraction of element *i* that is leached away during alteration. Based on compilations of exposed plutonic belts through time, Cao *et al*.^23^ reconstructed past continental arc lengths and report an excess continental arc length *L* of ~25,000 km in the Cretaceous. We then assume that the average width *w* of a continental magmatic arc is ~50–100 km and typical magmatic fluxes $\dot{M}$ for continental arcs are ~1 km/My^67^. The SiO_2_, FeO, and P_2_O_5_ concentrations of average continental arcs are 65, 3 and 0.2 wt.%^61,62^ while the mass fractions ${M}_{i}^{L}/{M}_{i}^{o}$ of Si, P, and Fe leached are 25–75% based on this study. The fraction of magmatic flux that gives rise to explosive eruptions, *f*_*ash*_, which generates ash, is poorly known. However, detailed studies of extrusive volcanic deposits and constraints on pluton size suggest that the intrusive to eruptive ratio in silicic magmatic systems is ~10:1, that is, ~10% of the magmatic flux is erupted^68^. What fraction of the erupted flux is explosive and generates ash is not known. We assume only 10% of the extrusive flux gives rise to ash, that is, ~1% of the *total* magmatic flux is ash. This estimate of the ash flux is a conservative estimate of the nutrient flux from Cretaceous continental arcs. Where we do have eruptive estimates, such as from the 0.76 Ma Bishop Tuff eruption of Long Valley caldera (CA, USA), which generated ~700 km^3^ of ash from a circular shaped magma body of ~5400 km^3^ (radius ~15 km and thickness ~8 km), 5–10% of the magma body was erupted in just one ash event (after correcting for density)^69^. In the above, one of the most important assumptions we have made is that the magmatic flux through an active continental arc is constant, resulting in the global magmatic and nutrient inputs scaling linearly with excess continental arc length. Whether this assumption represents an adequate approximation of how arcs operate on a global scale is unclear. In any case, weathering of volcanic crust in the arc itself, which we do not accounted for in this analysis, is likely to be large^70,71^, so our estimates of ash-laden nutrient inputs are minimum bounds on the global volcanic-derived nutrient inputs into the ocean.

**Figure 4:**
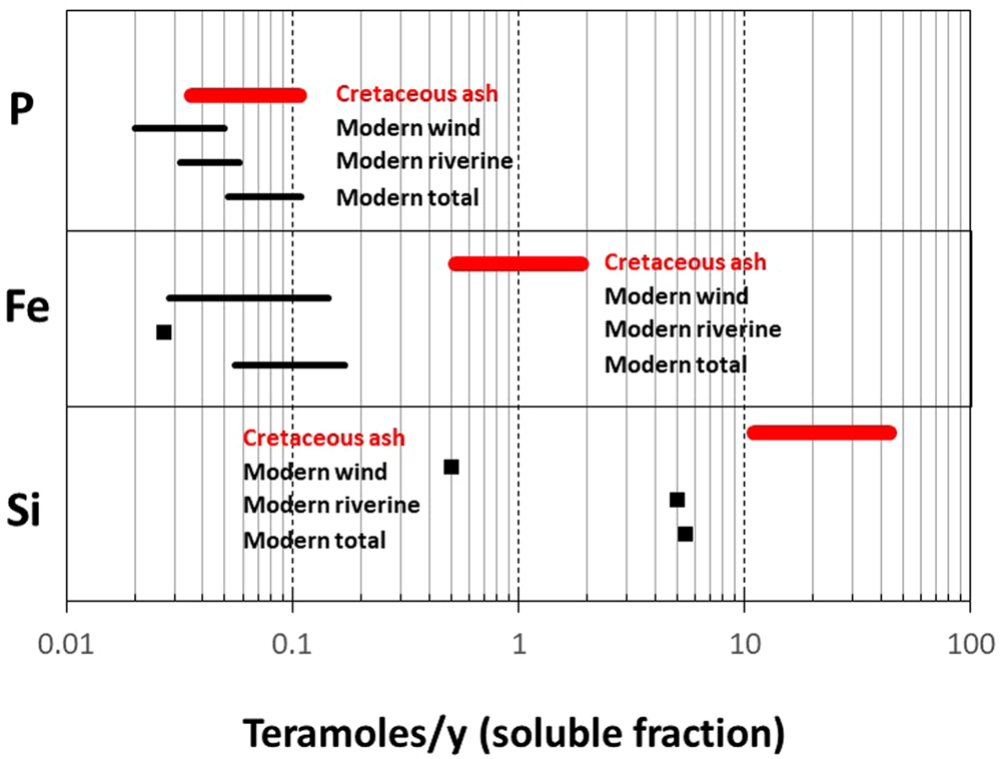
Estimated inputs of soluble Si, Fe and P in Teramoles/y. Red bars represent soluble flux from Cretaceous windblown ash only (this study). Black lines or squares represent soluble inputs into ocean for wind, riverine and combined riverine + wind in the present day (P from Table 3 in Ruttenberg^77^; Fe from Jickells *et al*.^76^ and Meybeck^73^; Si from Treguer *et al*.^72^ and Meybeck)^73^. Details of calculations are discussed in the text.

Based on the above calculations, we estimate time-averaged oceanic inputs of Si, Fe and P by windblown ash alone to be 11–44, 0.5–1.9 and 0.04–0.1 Tmoles/y during the Cretaceous (Fig. 4). For comparison, modern global (riverine + windblown) inputs of soluble Si into the ocean are ~5 Tmoles/y^72,73^, with >90% of the Si input to oceans coming from riverine flow and the remainder from windblown dust. Estimates of modern global inputs of soluble Fe into the ocean are highly uncertain because the soluble fraction of windblown Fe is not well constrained. Assuming the soluble fraction of windblown Fe ranges up to 10–50%^74,75^, the modern global Fe input to the oceans can range up to 0.055–0.17 Tmoles/y, with dust making up 50–80% of the global input^73,76^. If the soluble fraction of dust is only ~1% as in the case of soil Fe, global inputs would be much smaller. We consider these modern estimates of global Fe input to the ocean to be upper bounds. Modern global inputs of soluble P into the ocean range from ~0.05–0.1 Tmoles/y, with >50% coming from rivers and the remainder from windblown dust^77^.

Our results show that, during the Cretaceous, the soluble flux of Si, Fe and P *from windblown ash alone* was >2 times, >5 times and equal to or greater than the modern *global* inputs of soluble Si, Fe and P, respectively, into the oceans via windblown dust and riverine flow. The Cretaceous flare-up of continental arcs was also associated with enhanced erosion^67^, which would have also contributed to the inputs of Si, Fe and P via chemical weathering. The global (riverine and ash) input of Si, Fe and P into the Cretaceous oceans must therefore have been substantially higher than today.

## Implications

In the vast expanses of the modern open ocean, where there is limited upwelling, biological productivity is nutrient-limited, particularly in terms of Fe and P^52^. Our work here suggests biological productivity could have increased during the Cretaceous due to the more than doubling of continental arc volcanic activity, which would have increased global nutrient fluxes into the ocean by windblown ash deposition, increasing the global delivery of nutrients into the oceans. Distal shelf or slope environments in the Cretaceous oceans, which were otherwise starved of siliciclastic derived nutrients and probably too shallow to undergo extensive upwelling, appear to have experienced high sedimentation rates owing to high biogenic sediment accumulation^56^. Enhanced nutrient delivery via ash may have thus increased the efficiency of organic C production and preservation. This would predict that the fraction of volcanic CO_2_ inputs into the ocean/atmosphere, which was converted to organic carbon would have been higher in the Cretaceous. This prediction is consistent with the anomalously heavy carbon isotopic signature of Cretaceous seawater, which is widely thought to reflect a higher fraction of organic carbon *f*_*org*_ burial^78^, but the driving mechanism for the high *f*_*org*_ has been debated^78–80^. We suggest that the high quantities of hydrocarbon source rocks in the Cretaceous^81^ may be due to a combination of enhanced geologic inputs of carbon into the atmosphere through continental arc volcanism^16,23–26^ and sequestration of organic carbon driven by ash fertilization. We note that time intervals of intense organic carbon burial are often associated with global oceanic anoxia events, many of which occur in the Cretaceous or Paleogene^11^. Flood basalts have been suggested to be responsible for at least some of the oceanic anoxic events^82^, but the relationship to flood basalts may not be so simple because anoxia seems to also occur in lacustrine environments, which would be unaffected by nutrients delivered by flood basalts. However, what seems to be clear is that the background ocean system must have been primed in such a way that small environmental perturbations could trigger an anoxic event. It is generally agreed that ocean oxygen content may have been lower in the Cretaceous due to elevated temperatures imparted by higher atmospheric pCO_2_^11^, the latter driven by the higher volcanic inputs of CO_2_ during that time^16,23,24^. We speculate that enhanced ash inputs into both marine and terrestrial environments could have further pushed oceanic and lacustrine systems towards lower oxygen contents.

Our study predicts that high total organic carbon in sediments may at times be temporally and even spatially correlated with ash. Indeed, bentonite layers appear to be abundant in other source rocks, such as the Jurassic/Cretaceous Vaca Muerta in Argentina^83^ and the Cretaceous Niobrara formation in the United States^84^. Natural gamma ray logs of the Eagle Ford show that total organic carbon qualitatively correlates with Th and U concentration^53,85^, elements which likely derive originally from the ash and are then are leached and ultimately bound to organic carbon. In addition, Zr concentrations appear to correlate with sulfur content, the former deriving from ash and the latter an indirect measure of organic carbon^86^. In fact, altered ash is so commonly associated with shale source rocks that their presence is well known to interfere with drilling and hydrocarbon recovery^87^. In the Eagle Ford, sections with the highest organic carbon content appear to also have the most bentonite beds^56^.

While there are clearly other mechanisms for globally enhancing organic carbon burial, such as fertilization by large igneous provinces^88,89^, enhanced coastal upwelling by increasing continental margin length during supercontinent break-up^90^, or enhanced orogenic erosion^91^, the results of this study suggest that subduction-related volcanism, particularly continental arcs, can play an important role in the long-term carbon cycle^16–18,24,92^, not just through amplifying the inputs of volcanic CO_2_, but also in terms of nutrient delivery via ash. Further testing of the ash-organic carbon connection will require highly detailed chemo- and litho-stratigraphy in the context of igneous petrology as well as analyses of different sections in time and space. There is also a need to better understand how eruptive flux varies with different types of volcanism (composition and explosivity) and how ash is transported in the atmosphere, deposited in marine and terrestrial environments, and ultimately assimilated into the biosphere. Finally, more study is needed to evaluate the relative contributions of dissolution of ash in the marine environment and weathering of ash in terrestrial environments.

## Methods

Major and traces were determined by laser ablation inductively coupled plasma mass spectrometry (ICP-MS) using a 213 nm laser (New Wave) and a Thermo Element 2 ICP-MS following methods described in^93^. Analyses were done on 150 µm thick sections with a 100 µm diameter spot size at 5 Hz and a fluence of 3 J/cm^2^. The ICP-MS was operated in medium mass resolution mode (m/Δm~3000) in order to resolve various molecular interferences. We used multiple standards for external calibration (USGS BHVO-2g, BIR-1g, and BCR-2g). Sensitivity under these conditions ranged from 5,000–10,000 cps/ppm depending on which element was analyzed. We used ^30^Si and ^44^Ca as internal standards for carbonate-poor and carbonate-rich samples.

## Electronic supplementary material

Dataset 1 and 2
